# Saturation of an Intra-Gene Pool Linkage Map: Towards a Unified Consensus Linkage Map for Fine Mapping and Synteny Analysis in Common Bean

**DOI:** 10.1371/journal.pone.0028135

**Published:** 2011-12-08

**Authors:** Carlos H. Galeano, Andrea C. Fernandez, Natalia Franco-Herrera, Karen A. Cichy, Phillip E. McClean, Jos Vanderleyden, Matthew W. Blair

**Affiliations:** 1 Department of Microbial and Molecular Systems, Centre of Microbial and Plant Genetics, K.U. Leuven, Heverlee, Belgium; 2 Sugarbeet and Bean Research Unit, Agricultural Research Service, United States Department of Agriculture, East Lansing, Michigan, United States of America; 3 International Center for Tropical Agriculture (CIAT) Bean Project, Cali, Colombia; 4 Genomics and Bioinformatics Program, North Dakota State University, Fargo, North Dakota, United States of America; 5 Department of Plant Sciences, North Dakota State University, Fargo, North Dakota, United States of America; Nanjing Forestry University, China

## Abstract

Map-based cloning and fine mapping to find genes of interest and marker assisted selection (MAS) requires good genetic maps with reproducible markers. In this study, we saturated the linkage map of the intra-gene pool population of common bean DOR364×BAT477 (DB) by evaluating 2,706 molecular markers including SSR, SNP, and gene-based markers. On average the polymorphism rate was 7.7% due to the narrow genetic base between the parents. The DB linkage map consisted of 291 markers with a total map length of 1,788 cM. A consensus map was built using the core mapping populations derived from inter-gene pool crosses: DOR364×G19833 (DG) and BAT93×JALO EEP558 (BJ). The consensus map consisted of a total of 1,010 markers mapped, with a total map length of 2,041 cM across 11 linkage groups. On average, each linkage group on the consensus map contained 91 markers of which 83% were single copy markers. Finally, a synteny analysis was carried out using our highly saturated consensus maps compared with the soybean pseudo-chromosome assembly. A total of 772 marker sequences were compared with the soybean genome. A total of 44 syntenic blocks were identified. The linkage group Pv6 presented the most diverse pattern of synteny with seven syntenic blocks, and Pv9 showed the most consistent relations with soybean with just two syntenic blocks. Additionally, a co-linear analysis using common bean transcript map information against soybean coding sequences (CDS) revealed the relationship with 787 soybean genes. The common bean consensus map has allowed us to map a larger number of markers, to obtain a more complete coverage of the common bean genome. Our results, combined with synteny relationships provide tools to increase marker density in selected genomic regions to identify closely linked polymorphic markers for indirect selection, fine mapping or for positional cloning.

## Introduction

A linkage map indicates the position and relative genetic distances between markers along chromosomes and is based on the principle that genes and markers segregate via chromosome recombination during meiosis [1]. Therefore, genes or markers that are close or tightly-linked will be transmitted together from parent to progeny more frequently than genes or markers that are located further apart. Genetic linkage maps are an essential prerequisite for studying the inheritance of both qualitative and quantitative traits, to develop markers for marker assisted selection (MAS), for fine mapping and map-based cloning of genes of interest, and for comparative genomic studies. However, the utility of the linkage map information is often limited to the genetic background of the mapping population.

In common bean (*Phaseolus vulgaris* L.), the first linkage maps were developed with small numbers of linkage groups and included genes controlling mostly morphological and pigmentation traits such as flower and seed color or seed pattern [2], [3]. The advent of DNA based markers, restriction fragment length polymorphism (RFLP) and random amplified polymorphic DNA (RAPD), amplified fragment length polymorphism (AFLP), and simple sequence repeats (SSR) [4], [5], led to more detailed maps.

The first integration of three separate linkage maps used the recombinant inbred population BAT93×Jalo EEP558 as the core map [6]. Subsequently, a SSR linkage map of the population DOR364×G19833 was integrated with the BAT93×Jalo EEP558 map [7]. Since then, both populations (BAT93×Jalo EEP558 and DOR364×G19833) have been used by different research groups for map saturation using SSR [8]–[10] and SNP markers [11], [12], as well as for QTL identification [13]–[18], physical mapping [19], [20] and gene-based marker evaluation [12], [21], [22].

The construction of a consensus map combining the information of multiple segregating populations from diverse genetic backgrounds, offers the opportunity to map a larger number of loci than in most single crosses, thus increasing the number of potentially useful markers across divergent genetic backgrounds and providing greater genome coverage, in addition to providing opportunities to validate marker order [23]. The consensus map captures more markers, genes or QTL than could be mapped in a single population study due to limited marker and phenotypic polymorphisms found within a single population [24]. For common bean, a consensus map would collate loci discovered using populations developed within Mesoamerican [25]–[27] or Andean gene pools [28], where it has only been possible to develop low density maps because of low polymorphism rates.

Consensus maps have been developed in several crops using different methodologies such as a visual approach in wheat (*Triticum aestivum* L. em. Tell) [29] or pooling the marker data of different mapping populations of maize (*Zea maize*) to generate a “pooled map” [30]. The software JoinMap [31] weights pairwise genetic distances based on population structure and size, and has become a very popular consensus map tool in several crops like soybean *Glycine max* [32], rye (*Secale cereale* L.) [33], melon (*Cucumis melo* L.) [34] and cotton (*Gossipum* spp.) [35].

Graph theory is now being utilized as an approach to identify the most accurate consensus map [24], [36]. The map is modeled as a directed acyclic graph (DAG) in which nodes represent mapped markers and edges define the order of adjacent markers. Based on shared vertices, DAGs are merged into a consensus map. Earlier this year, MergeMap software was developed [37], where the order of conflicts or cycles are resolved parsimoniously, an approach that showed improved performance in terms of accuracy and run time when compared to other programs. This software has been successfully used for the construction of a consensus map from six populations based on 1,375 SNP markers in cowpea *Vigna ungliculata* [38] and for three mapping populations with 2,943 SNP markers in barley (*Hordeum vulgare*) [39].

Synteny analysis is the comparison of genetic maps between species rather than between populations and usually requires whole genome sequences. In terms of legume genomics, soybean, medicago (*Medicago truncatula*) and lotus (*Lotus japonicus*) are three legumes that have complete or almost complete genome sequence information. These genome sequences have been useful to compare genomes, and to transfer information from genome sequence information to other crop species. However, the ability to transfer knowledge between species depends on both the evolutionary distance between species, and the rate and nature of changes in the genome over time [40].

Therefore, analyses that have compared common bean and sequenced legumes reported syntenic blocks of various sizes [12], [21], [22], [41]. However, in these studies the common bean information came from low or medium saturated linkage maps developed using a single bi-parental population. Here, we report the saturation of the linkage map from a Mesoamerican population, DOR364×BAT477, using SSR and gene based markers, followed by comparisons to the inter-gene pool linkage maps of the crosses DOR364×G19833 and BAT93×JALO EEP558 to finally build a saturated, consensus map. Additionally, the consensus map was compared with the genome of the soybean. Syntenic relationships were defined which provide *in silico* evidence for the position of new markers that can be used for fine mapping projects and positional cloning.

## Materials and Methods

### Plant material

The population DOR364×BAT477 consists of 113 F5:7 recombinant inbred lines (RILs) as described in [27]. For map saturation, the first 92 lines were selected. The DNA of the population and the parents was extracted using 5 g of tissue as described in [42]. The extraction quality was checked on 1% agarose electrophoresis, and the DNA was quantified with Quantity One® v 4.0.3 software (Bio-Rad) using a DNA lambda ladder as a size reference. Finally, DNA was diluted to a final concentration of 5 ng/µl.

### Map saturation

The parental genotypes were evaluated with 2,706 common bean DNA based markers including SSRs based on EST libraries and BAC end sequences, as well as gene-based markers from a total of 24 sources of markers (Table S1). Among these, the legume anchor markers (LEG) reported by [21] were evaluated in both the DB and in an additional population, DOR364×G19833, as described below. The electrophoresis and PCR parameters for SSR and gene based markers were as described previously [7], [12 respectively]. Polymorphic markers were then evaluated on the entire DB mapping population. The linkage groups were named after previous reports [22], [41].

### Linkage analysis

Segregation data was used to place the new markers on the DOR364×BAT477 population linkage map described in [27]. Linkage analysis was conducted with the Kosambi mapping function using the software application Mapmaker 2.0 for Windows [43]. The markers were placed to the established linkage groups with the ‘try’ and ‘compare’ commands with a minimum LOD of 4.0. All linkage maps were drawn using MapChart [44].

### Consensus map

The core mapping populations were derived from inter-gene pool crosses: DOR364×G19833 (DG; n = 87) and BAT93×JALO EEP558 (BJ; n = 79). These were used to build a consensus map with the less saturated DOR364×BAT477 population (DB). The DG linkage map was developed by CIAT Bean Project [7], [10]–[12], [19], [20]. The BJ linkage map was developed using reported map information [21], [22]. The consensus map was constructed with MergeMap [37]. The consensus map coordinates from MergeMap were normalized to the arithmetic mean cM distance [39] for each linkage group using data reported for the three individual maps. The consensus map and the relationships with the single linkage maps were drawn using MapChart [44].

### Synteny analysis

The first genomic synteny analysis was conducted using a total of 772 marker sequences from the consensus map, downloaded in FASTA format from NCBI and compared with the soybean (version Glyma1) genome sequence following the methodology reported by [12] with some modification. The common bean sequences were aligned against the chromosome based assembly of soybean using local blastn. Graphics were drawn with MapSynteny, an in-house software created with Visual Basic Script programming language in a Microsoft Excel™ environment (available upon request from the corresponding authors). The genic synteny analysis was carried out by aligning the marker sequences against the public common bean EST assembly from Bean Gene Index (Dana-Farber Cancer Institute - DFCI) (March 24, 2011). A total of 491 tentative consensus (TC) sequences were aligned against the coding sequences (CDS) of soybean, with the same blast parameters described above. The relationships of the homeologous segments within the soybean genome were then drawn with Circos software version 0.54 [45].

## Results

### Parent marker survey

At the beginning of this study, the DOR364×BAT47 linkage map consisted of 186 markers, linked by 60 SSRs and 126 dominant AFLP or RAPD markers [27]. With the aim of increasing the marker saturation in this linkage map, a total of 2,706 markers were evaluated between the parents DOR364 and BAT477, including 1,136 genomic SSR, 866 genic SSR and 393 gene-based markers (Table S1). Averaged over all markers, the polymorphism rate was low at 7.7% with monomorphism for several sets of markers [46]–[49].

The polymorphism frequency was higher in genomic than in genic SSR. A polymorphism rate higher than 10% was obtained for genomic SSR reported by [9], [10], [50]. Interestingly, the SSR markers developed by Buso et al. [51] had the highest polymorphism rate of 40%. In contrast, few polymorphisms were found for genic SSR. The most polymorphic genic SSRs were the set developed by Hurtado (unpublished) with a polymorphism rate of 6.6%. On average, the polymorphism rate for the DB population was 3.6% for genic SSRs and 10.7% for genomic SSRs. The same low polymorphism rate was found with gene based markers using the single-strand conformation polymorphism (SSCP) technique. On average, the polymorphism frequency was 1.6%. In summary, 111 new markers comprising 100 SSR markers and 11 gene-based markers were polymorphic and were mapped along with 120 of the dominant markers originally used in the previous analysis with the DB population [27].

### Segregation analysis

**Figure 1:**
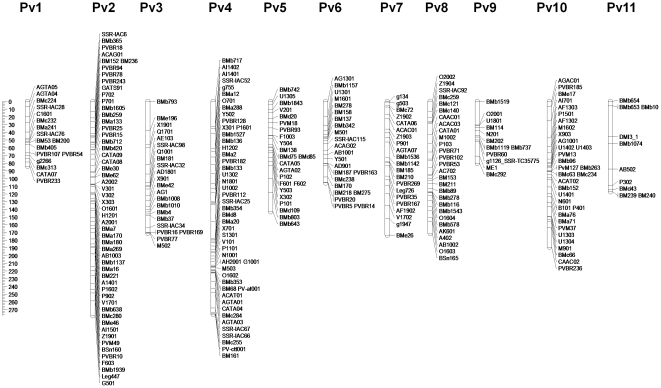
Linkage map based on recombinant inbred lines of the intra-gene pool population DOR364×BAT477. Map was constructed using MAPMAKER/EXP 3.0 with Kosambi mapping function. The bar on the left hand side shows the distance in centiMorgans (cM) from the top of each chromosome.

A new DB map was developed by incorporating these 111 markers with the previous segregation analysis of 180 markers [27]. A total of 291 markers were placed in the linkage map, including AFLP, RAPD, SSR and gene-based markers (Figure 1, Table 1). The SSR and RAPD were the most abundant markers in the linkage map, with 160 and 98 markers, respectively. Specifically, 74% of the Pv1 markers were SSRs. The total map length was 1,789 cM and linkage group size ranged from 80 cM (Pv9) to 277 cM (Pv4) with an average of 163 cM per linkage group (Figure 1, Table 1).

**Table 1:** Linkage map summary information for the DOR364×BAT477 population.

LG	AFLP	RAPD	SSR	BES_SSR	EST_SSR	Gene-based marker	Total markers	Distance cM	cM between markers
Pv1	3	1	11	1		1	17	84.05	4.94
Pv2	3	18	20	8	3	2	54	277.81	5.14
Pv3		7	8	5	2		22	170.97	7.77
Pv4	4	19	18	6		1	48	276.16	5.75
Pv5	2	10	7	4			23	142.92	6.21
Pv6	1	7	13	2			23	105.79	4.60
Pv7	3	5	6	2	1	4	21	173.59	8,27
Pv8	3	10	9	5		1	28	171.18	6.11
Pv9		3	5	3		1	12	80.30	6.69
Pv10	3	16	10	3	1		33	188.88	5.72
Pv11		2	3	4		1	10	117.00	11.70
**Total**	**22**	**98**	**110**	**43**	**7**	**11**	**291**	**1,788.66**	**6.15**

In general, the marker loci were well distributed within the linkage groups with an average of 26 markers per linkage group. The number of marker loci per linkage group ranged from 10 on Pv11 to 54 on Pv02. The average distance between markers was 6 cM, ranging from 4.6 cM on Pv6 to 8.4 cM on Pv7. Based on Chi square tests (P<0.05), segregation distortion was found at the top of linkage group Pv4 which represented preferential transmission of the DOR364 allele. Some gaps greater than 20 cM were still present in linkage groups Pv3, Pv4, Pv5, Pv7, Pv9, Pv10 and Pv11, despite the addition of the new markers.

### Consensus map

Due to the low polymorphism rate found in the DB population, a consensus map was developed in order to increase the marker saturation and to improve the marker order. The DG and BJ mapping populations along with the DB population were used to build the consensus map. The DG map, developed by the CIAT bean genetics program, consisted of 499 single copy markers, including 31 RFLP, 141 SNP, 322 SSR and 5 STS. The map was 2,306 cM, with an average linkage group size of 209 cM and with average marker density of one marker per 4.6 cM. The BJ linkage map used here consisted of 424 markers, including 21 SSR, 20 RFLP, 381 SNP, one RAPD and one phenotypic marker. The full map length was 1,991 cM, with an average linkage group size of 180.9 cM and an average density of one marker per 4.6 cM.

**Figure 2:**
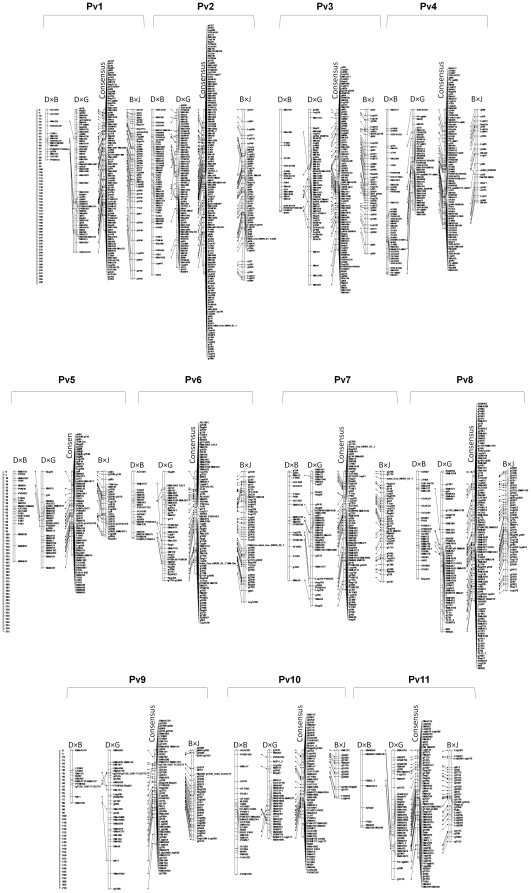
Common bean consensus map from three mapping populations represented by 1010 mapped loci covering 11 linkage groups. Map distances are shown in cM as a ruler at the left hand side. The linkage groups belonging to populations DOR364×BAT477, DOR364×G19833 and BAT93×JALO EEP558 are identified with the letters DB, DG and BJ, respectively. Loci that are common between pairs of populations are connected by lines.

**Figure 3:**
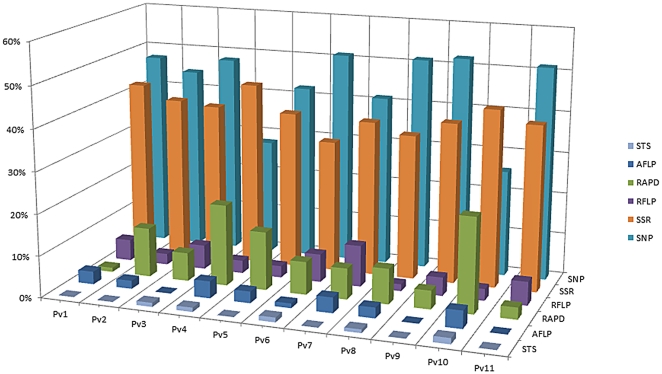
Types of markers and total proportion of markers used in the consensus map.

The consensus linkage map developed with information from the 257 RILs of the three populations is shown in Figure 2. A total of 98, 87, 14 and 4 common anchor markers are shared between DG-DB, DG-BJ, DB-BJ and DG-BJ-DB, respectively (Table 2). On average, each linkage group shared 18 anchor markers with a range from 39 (Pv2) to 7 (Pv5) (Table 2). In total 1,010 markers were placed in the consensus map, including 446 SNP, 392 SSR, 99 RAPD, 45 RFLP, 22 AFLP, 5 STS and one phenotypic marker (Figure 3). On average the consensus maps consisted of 91 markers per linkage group with a maximum of 151 on linkage group Pv2 and a minimum of 67 on the linkage group Pv9. The total full map length was 2,041 cM while linkage groups ranged in size from 131 cM (Pv10) to 276 cM (Pv2) with an average of 185 cM per linkage group. The average distance between markers was 2 cM, and the largest gaps were of 21 and 25 cM in linkage groups Pv9 and Pv4, respectively. Moreover, even though marker order among the four maps (consensus, DB, DG and BJ) was reliable, some slight differences were observed between the consensus and single maps (Figure 2).

**Table 2:** Consensus genetic map summary and the synteny relationship with model legumes.

LG	Anchor markers				Total markers	Distance cM	AVG between markers	Syntenic blocks	
	DB-BJ	DG-BJ	DG-DB	DB-DG-BJ				Gm chromosomes*	Orthologous loci
Pv1	1	10	9		97	202.52	2.09	14/17,3/19,11/18	88
Pv2	2	14	23		151	276.36	1.83	1/11, 1/2, 1/9, 8/5	121
Pv3		12	10		102	235.77	2.31	2/16, 17/5, 17/2, 17/7, 17/13, 16/8	86
Pv4	2	2	13	1	97	199.87	2.06	9/7, 16/9, 16/2, 13/19	51
Pv5	1	3	3		71	132.93	1.87	13/12, 13/15, 8/15	64
Pv6	1	12	8	1	88	162.48	1.85	18/11, 18/8, 15/8, 12/8, 19/3, 15/9, 15/13	65
Pv7	4	8	7	2	80	187.87	2.35	20/10, 13/10, 2	57
Pv8	1	10	6		117	205.49	1.76	18/8, 18/2, 18/9, 18/7, 2/14	97
Pv9	2	7	5		67	142.48	2.13	6/4, 9/15	63
Pv10		3	9		70	131.88	1.88	7/8, 7/16, 3/1, 3/7	44
Pv11		6	5		70	163.79	2.34	12/11, 12/6, 15/13	51
**Total**	**14**	**87**	**98**	**4**	**1010**	**2,041.44**	**2.02**		787

*The chromosomes with “/” means the soybean duplicated chromosome.

The SNPs and SSRs markers were well distributed throughout the linkage groups. However, in general the SNP markers were more frequent, with the exception of the LG Pv4 and Pv10 where the SSR markers were more frequent (Figure 3). In linkage groups Pv6, Pv08, Pv9 and Pv11, more than 50% of the markers were SNPs.

### Synteny analysis with soybean

A total of 772 marker sequences distributed in the common bean consensus map were aligned with the soybean 1.01 genome [52]. The soybean genome is thought to be based on two duplications that occurred approximately 59 and 13 million years ago, resulting in homeologous relationships between segments of the 20 soybean chromosomes [52]. Therefore, two highest hits were selected for the synteny analysis [12], [41]. As such, 506 and 470 soybean orthologous sequences were identified with the first and second hit, respectively.

**Figure 4:**
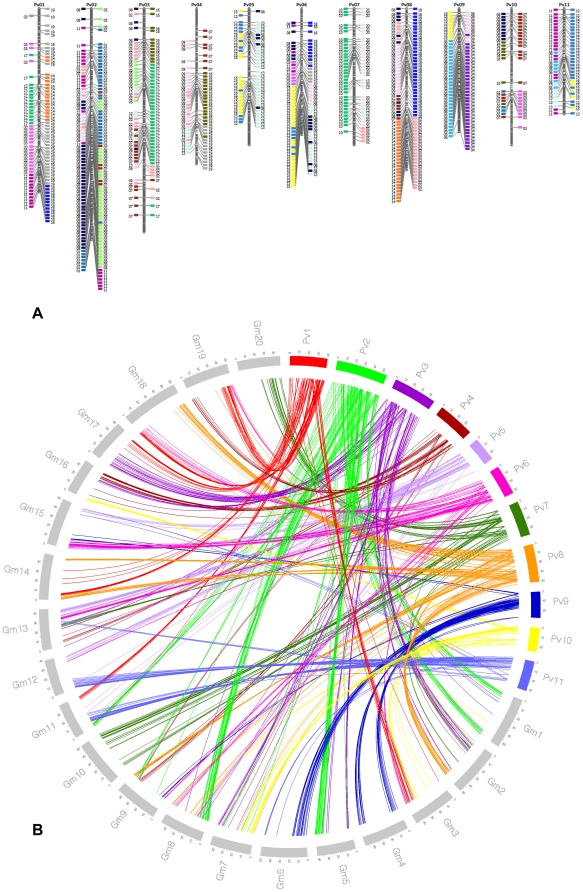
Synteny relationships between common bean and soybean. a). Associations between common bean and soybean linkage groups through sequence based markers are shown. The colored boxes represent the homologies with chromosome segments from the soybean genome with each chromosome from soybean assigned a given specific color. The boxes to the right side of the linkage group are the first similarity matches, while to the left side are the second similarity matches. b). Schematic representation of the genic synteny relationship of the common bean transcript map with the CDS of soybean. Each line represents the direct relationship with a specific soybean gene.

The difference between the number of identified sequences for the first and second hits was because the second hit sometimes did not meet the e-value threshold. The most syntenic loci were found on Pv2, with 156 orthologous sequences, whereas Pv10 had the fewest loci with 50 only. On average, 88 hits were found per linkage group. A total of 87 synteny groups were found corresponding to 44 common bean regions (Table 2, Figure 4a). The linkage group Pv6 contained seven syntenic blocks while Pv9 only contained two. Some syntenic gaps were noted at the top of the linkage group Pv4 and Pv6 and at the end of Pv3 and Pv10 (Figure 4a).

Using transcript information, a total of 491 common bean TC sequences were also compared against soybean CDS sequences. A total of 405 and 382 soybean genes were identified in the first and second hit, respectively. On average 71 genes per linkage group were found, ranging from 121 genes on linkage group Pv2 to 44 on linkage group Pv10. Figure 4b represents the collinear gene blocks among 20 soybean chromosomes and 11 linkage groups in common bean. The five most saturated syntenic blocks were the Gm8/Gm5 with 58 genes on Pv2, Gm6/Gm4 with 40 genes on Pv9, Gm20/Gm10 with 36 genes on Pv7, Gm12/Gm11 with 32 genes on the Pv11 and Gm2/Gm14 with 30 genes on the Pv08 (Table 2).

## Discussion

### Linkage map saturation

The first objective of this study was to saturate the linkage map of the intra-gene pool population DB. However, marker screening in the parents revealed a low polymorphism rate. The low polymorphism reported here was consistent with the results using AFLP, RAPD and SSR in the construction of the original DB framework map [27]. On average, the genomic SSR polymorphism rate was 9.5%, a lower rate than observed for other intra-gene pool populations. Low to medium polymorphism rates were found using the Mesoamerican population BAT 881×G21212 (30%) [26] and 31% using the Andean population G19833×AND696 [28].

In contrast, using inter-gene pool populations, researchers have reported polymorphism rates of 56% for the DG population [7] and 42% and 55.7% for the BJ population [7], [9]. In addition, higher polymorphism rates were reported for the DG population using other markers [10], [20]. The low polymorphism reported here could be explained by the fact that the genotypes DOR364 and BAT477 belong to the Mesoamerican gene pool and also belong to the Mesoamerican race, thus showing less polymorphism compared with other intra-gene pool populations developed from members of different races [53]. However, despite this narrow genetic base, these genotypes exhibit contrasting physiological behavior in key agricultural traits like drought [27], low phosphorus stress and symbiotic nitrogen fixation [25].

### Consensus map

Efforts to compare linkage maps in common bean based on RFLP and SSR markers were reported previously in integrated mapping by [6], [7]. Here we report the first consensus map in common bean built from a Mesoamerican intra-gene pool and inter-gene pool (DG, BJ) populations. The consensus map was created using MergeMap [37], which has recently been used for other species [38], [39], [54]. Other approaches have been used in the past to construct consensus maps, most commonly using the JoinMap software [31]. Both methodologies were compared using the same set of data [37], and Mergemap was found to be more accurate in terms of marker order, and significantly faster than JoinMap. Similar comparisons reported that Mergemap appeared to outperform Joinmap in terms of marker order consistency between integrated maps [54]. However, Joinmap tended to produce more accurate estimates of genetic distances. Another drawback of Joinmap is that when using linkage maps generated by MapMaker software changes in markers order and distances were observed. JoinMap uses all pairwise estimates, above the defined LOD threshold, to establish map length, whereas MapMaker establishes map length using only adjoining marker pairs to calculate the sum of adjacent distances [33].

The common bean consensus map exhibits a higher marker density than previous linkage maps reported for bi-parental populations. Maps based on the DG population with 280 [19] and 288 [12] markers have been reported. Likewise, using the BJ population, 275 markers have been placed on the common bean genetic map [22]. Here, a consensus map with nearly thousand markers distributed on 11 linkage groups with a mean distance of 2 cM between adjacent loci was developed. In terms of marker order, the consensus map had few changes as compared to the individual maps. These small differences could be explained by different recombination events among population parents, small progeny size in any single population, and a generally increased recombination rate in terminal regions of linkage groups [55], [56].

Therefore, the consensus marker order is significantly more reliable, because a much higher number of individuals and higher number of recombination events was taken into account when combining the three populations. Similar results were reported when a consensus map was developed for grape (*Vitis vinifera* L.) based on three populations [55]. Also, a consensus map using three populations of *Brassica napus* producing a highly saturated map with 5,162 genetic markers [54]. In addition, the length of our consensus map is 2,041 cM, slightly higher than single maps of DB and BJ populations and previous maps reports [6], [7], [8], [9], [27], [28]. Consensus maps with increased map size have been reported with other species [23], [55], [56]. Part of this increase may be due to an improved coverage of the ends of the chromosomes [56].

In our consensus map two gaps greater that 20 cM remain. These areas of low marker density may correspond to genomic regions of similar ancestry or identity by descent in the populations used in this study. Similar gaps were obtained in the consensus map of sorghum (*S. bicolor* L.) [23] with low polymorphism and that were identical by descent.

### Synteny relationship

The large and consistent synteny blocks reported here resulted from an extended consensus map based on mapping information from three mapping studies in common bean [12], [21], [41]. The syntenic groups identified here (Table 2) are consistent with the previous reports and allow us to extend the syntenic analyses of these two species, as well as to confirm homeologous segment analysis in soybean that has been extensively reported on. Interestingly, almost the entire Pv7 linkage group showed a strong relationship with the syntenic block Gm10/Gm20. These results are corroborated with soybean genome analysis where chromosome 20 is highly homologous to the long arm of chromosome 10 [52] suggesting that Gm10, Gm20, and Pv7 are good candidates to identify ancestral chromosomal duplication of legume genomes.

Another good candidate for evolutionary genomics is the linkage group Pv9 that showed very strong relationships with the synteny blocks Gm6/Gm4 and Gm15/Gm19. That the one-two relationship does not extend over the entire Pv chromosomes further supports the conclusion by McClean et al. [41] that the large scale order of soybean chromosomes is the result of chromosome breakage/union events possibly directly associated with the tetra-ploidization event in the genome history of soybean.

Synteny-based analysis in cereals has allowed the identification of seven shared duplications which led to the modeling of a common ancestral genome structure of 33.6 Mb structured in five protochromosomes containing 9,138 protogenes. This type of analysis provided new insights into the evolution of cereal genomes from their extinct ancestors [57] and this approach provides a reference tool for improved gene annotation and cross-genome marker development.

### Common bean breeding application

A consensus map in common bean increases the genome coverage and makes it possible to compare locations of major genes controlling important phenotypic traits or QTL positions between populations from multiple crosses. This is especially useful in populations with low recombination polymorphism, as the crosses within Andean or Mesoamerican gene pools, where genetic map saturation is difficult to obtain [27]. One of the uses of combining consensus maps with synteny relationships is to provide tools to increase marker density in selected genomic regions.

Such increases in marker density can be used to identify closely linked polymorphic markers for indirect selection, fine mapping or for map-based cloning. Examples of the advantage of the consensus map and their synteny analysis in other species have been recently reported in cereals. A meta-QTL analysis in sorghum (*Sorghum bicolor* L.) revealed that QTL and genes were located in heterochromatin regions [58]. In bread wheat (*Triticum aestivum* L.), a major nitrogen use efficiency (NUE) ortho-metaQTL is conserved at orthologous positions in wheat, rice, sorghum and maize [59]. In legumes, the consensus map in cowpea *V. unguiculata* was utilized for synteny based candidate gene identification and definition of QTL location for *Macrophomina phaselina* resistance [60].

Finally, given that the consensus map we have constructed for common bean contains more that 50% of the markers corresponding to coding regions this study provides an excellent functional framework for candidate gene dissection, expression network analysis, or analysis of legume genome evolution.

## Supporting Information

Table S1 **Summary of the markers evaluated in the DOR364×BAT477 (DB) population.**
(DOCX)

## References

[1] Collard B, Jahufer MZ, Brouwe JB, Pang EC (2005). An introduction to markers, quantitative trait loci (QTL) mapping and marker-assisted selection for crop improvement: The basic concepts.. Euphytica.

[2] Bassett MJ (1991). Five primary trisomics from translocation heterozygote progeny in common bean, *Phaseolus vulgaris* L.. Theor Appl Genet.

[3] Gepts P (1988). Provisional linkage map of common bean.. Annu Rep Bean Improv Coop.

[4] Nodari RO, Tsai SM, Guzman P, Gilbertson RL, Gepts P (1993). Towards an integrated linkage map of common bean. III. Mapping genetic factors controlling host-bacteria interactions.. Genetics.

[5] Vallejos CE, Sakiyama NS, Chase CD (1992). A molecular marker-based linkage map of *Phaseolus vulgaris* L.. Genetics.

[6] Freyre R, Skroch PW, Geffory V, Adam-Blondon AF, Shirmohamadali A (1998). Towards an integrated linkage map of common bean. 4. Development of a core linkage map and alignment of RFLP maps.. Theor Appl Genet.

[7] Blair M, Pedraza F, Buendia H, Gaitan E, Beebe S (2003). Development of a genome wide anchored microsatellite for common bean (*Phaseolus vulgari*s L).. Theor Appl Genet.

[8] Hanai L, Santini L, Camargo L, Fungaro M, Gepts P (2009). Extension of the core map of common bean with EST-SSR, RGA, AFLP, and putative functional markers.. Molecular Breeding.

[9] Grisi MCM, Blair MW, Gepts P, Brondani C, Pereira PAA (2007). Genetic mapping of a new set of microsatellite markers in a reference common bean (*Phaseolus vulgaris*) population BAT93×Jalo EEP558.. Genetics and Molecular Research.

[10] Blair MW, Buendía HF, Giraldo MC, Métais I, Peltier D (2008). Characterization of AT-rich microsatellites in common bean (*Phaseolus vulgaris L*.).. Theor Appl Genet.

[11] Galeano CH, Gomez M, Rodriguez LM, Blair MW (2009). CEL I nuclease digestion for SNP discovery and marker development in common bean (*Phaseolus vulgaris* L.).. Crop Science.

[12] Galeano CH, Fernandez AC, Gomez M, Blair MW (2009). Single strand conformation polymorphism based SNP and indel markers for genetic mapping and synteny analysis of common bean (*Phaseolus vulgaris* L.).. BMC Genomics.

[13] Liao H, Yan X, Rubio G, Beebe SE, Blair MW (2004). Genetic mapping of basal root gravitropism and phosphorus acquisition efficiency in common bean.. Funct Plant Biol.

[14] Beebe SE, Rojas-Pierce M, Yan X, Blair MW, Pedraza F (2006). Quantitative trait loci for root architecture traits correlated with phosphorus acquisition in common bean.. Crop Science.

[15] Blair M, Astudillo C, Grusak M, Graham R, Beebe S (2009). Inheritance of seed iron and zinc concentrations in common bean (*Phaseolus vulgaris* L.).. Molecular Breeding.

[16] Lopez-Marin HD, Rao IM, Blair MW (2009). Quantitative trait loci for root morphology traits under aluminum stress in common bean (*Phaseolus vulgaris* L.).. Theor Appl Genet.

[17] Caldas GV, Blair MW (2009). Inheritance of seed condensed tannins and their relationship with seed-coat color and pattern genes in common bean (*Phaseolus vulgaris* L.).. Theoretical and Applied Genetics.

[18] Blair MW, Sandoval TA, Caldas GV, Beebe SE, Páez MI (2009). Quantitative trait locus analysis of seed phosphorus and seed phytate content in a recombinant inbred line population of common bean.. Crop Sci.

[19] Córdoba JM, Chavarro C, Rojas F, Muñoz C, Blair MW (2010). Identification and mapping of simple sequence repeat markers from common bean (*Phaseolus vulgaris* L.) bacterial artificial chromosome end sequences for genome characterization and genetic-physical map integration.. Plant Genome.

[20] Córdoba JM, Chavarro C, Schlueter JA, Jackson SA, Blair MW (2010). Integration of physical and genetic maps of common bean through BAC-derived microsatellite markers.. BMC Genomics.

[21] Hougaard BK, Madsen LH, Sandal N, Moretzsohn MC, Fredslund J (2008). Legume anchor markers link syntenic regions between *Phaseolus vulgaris*, *Lotus japonicus*, *Medicago truncatula* and *Arachis*.. Genetics.

[22] McConnell M, Mamidi S, Lee R, Chikara S, Rossi M (2010). Syntenic relationships among legumes revealed using a gene-based genetic linkage map of common bean (*Phaseolus vulgaris* L.).. Theor Appl Genet.

[23] Mace E, Rami J-F, Bouchet S, Klein P, Klein R (2009). A consensus genetic map of sorghum that integrates multiple component maps and high-throughput Diversity Array Technology (DArT) markers.. BMC Plant Biology.

[24] Yap IV, Schneider D, Kleinberg J, Matthews D, Cartinhour S (2003). A graph-theoretic approach to comparing and integrating genetic, physical and sequence-based maps.. Genetics.

[25] Remans R, Beebe S, Blair M, Manrique G, Tovar E (2008). Physiological and genetic analysis of root responsiveness to auxin-producing plant growth-promoting bacteria in common bean (*Phaseolus vulgaris* L.).. Plant and soil.

[26] Frei A, Blair MW, Cardona C, Beebe SE, Gu H (2005). QTL mapping of resistance to *Thrips palmi* karny in common bean.. Crop Sci.

[27] Blair M, Galeano C, Tovar E, Muñoz Torres M, Castrillón A (2010). Development of a Mesoamerican intra-genepool genetic map for quantitative trait loci detection in a drought tolerant×susceptible common bean (*Phaseolus vulgaris* L.) cross.. Molecular Breeding.

[28] Cichy KA, Blair MW, Galeano CH, Snapp SS, Kelly JD (2009). QTL analysis of root architecture traits and low phosphorus tolerance in an Andean bean population.. Crop Sci.

[29] Marino CL, Tuleen NA, Hart GE, Nelson JC, Sorrells ME (1996). Molecular genetic maps of the group 6 chromosomes of hexaploid wheat (*Triticum aestivum* L. em. Thell.).. Genome.

[30] Beavis WD, Grant D (1991). A linkage map based on information from four F2 populations in maize (Zea mays L.).. Theor Appl Genet.

[31] Stam P (1993). Construction of integrated genetic linkage maps by means of a new computer package: JoinMap.. Plant J.

[32] Hyten, David L, Choi, Ik Y, Qijian S (2010). A high density integrated genetic linkage map of soybean and the development of a 1536 universal soy linkage panel for quantitative trait locus mapping.. Crop Sci.

[33] Gustafson J, Ma X-F, Korzun V, Snape J (2009). A consensus map of rye integrating mapping data from five mapping populations.. Theor Appl Genet.

[34] Cuevas H, Staub J, Simon P, Zalapa J (2009). A consensus linkage map identifies genomic regions controlling fruit maturity and beta-carotene-associated flesh color in melon (*Cucumis melo* L.).. Theor Appl Genet.

[35] Lacape J-M, Jacobs J, Arioli T, Derijcker R, Forestier-Chiron N (2009). A new interspecific, *Gossypium hirsutum*×*G. barbadense*, RIL population: towards a unified consensus linkage map of tetraploid cotton.. Theor Appl Genet.

[36] Jackson BN, Aluru S, Schnable PS (2005). Consensus genetic maps: a graph theoretic approach..

[37] Wu Y, Close TJ, Lonardi S (2011). Accurate Construction of Consensus Genetic Maps via Integer Linear Programming.. IEEE/ACM Transactions on Computational Biology and Bioinformatics.

[38] Muchero W, Diop NN, Bhat PR, Fenton RD, Wanamaker S (2009). A consensus genetic map of cowpea Vigna unguiculata (L) Walp. and synteny based on EST-derived SNPs.. Proc Natl Acad Sci USA.

[39] Close T, Bhat P, Lonardi S, Wu Y, Rostoks N (2009). Development and implementation of high-throughput SNP genotyping in barley.. BMC Genomics.

[40] Cannon SB, May GD, Jackson SA (2009). Three sequenced legume genomes and many crop species: rich opportunities for translational genomics.. Plant Physiol.

[41] McClean P, Mamidi S, McConnell M, Chikara S, Lee R (2010). Synteny mapping between common bean and soybean reveals extensive blocks of shared loci.. BMC Genomics.

[42] Tohme J, Gonzales D, Beebe S, Duque MC (1996). AFLP analysis of gene pools of a wild bean core collection.. Crop Sci.

[43] Lander E, Green P, Abrahamson J, Barlow A, Daly M (1987). MAPMAKER: an interactive computer package for constructing primary genetic linkage maps of experimental and natural populations.. Genomics.

[44] Voorrips RE (2002). MapChart: software for the graphical presentation of linkage maps and QTLs.. Journal of Heredity.

[45] Krzywinski M, Schein J, Birol Än, Connors J, Gascoyne R (2009). Circos: An information aesthetic for comparative genomics.. Genome Research.

[46] de Campos T, Benchimol LL, Moraes Carbonell SA, Chioratto AF, Fernandes Formighieri E (2007). Microsatellites for genetic studies and breeding programs in common bean.. Pesq agropec bras.

[47] Caixeta ET, Borém A, Kelly JD (2005). Development of microsatellite markers based on BAC common bean clones.. Crop Breeding and Applied Biotechnology.

[48] Yaish MWF, Perez de la Vega M (2003). Isolation of (GA)n microsatellite sequences and description of a predicted MADS-box sequence isolated from common bean (*Phaseolus vulgaris* L.).. Genetics and Molecular Biology.

[49] Guerra-Sanz JM (2004). Short Communication. New SSR markers of *Phaseolus vulgaris* from sequence databases.. Plant Breeding.

[50] Gáitan-Solis E, Duque MC, Edwards KJ, Tohme J (2002). Microsatellite Repeats in Common Bean (*Phaseolus vulgaris*): Isolation, Characterization, and Cross-Species Amplification in *Phaseolus* ssp.. Crop Science.

[51] Buso GC, Amaral ZS, Brondani RV, Ferreira ME (2006). Microsatellite markers for the common bean *Phaseolus vulgaris*.. Molecular Ecology Notes.

[52] Schmutz J, Cannon SB, Schlueter J, Ma JX, Mitros T (2010). Genome sequence of the palaeopolyploid soybean.. Nature.

[53] Blair M, Giraldo M, Buendia H, Tovar E, Duque M (2006). Microsatellite marker diversity in common bean (*Phaseolus vulgaris* L.).. Theor Appl Genet.

[54] Wang J, Lydiate D, Parkin I, Falentin C, Delourme R (2011). Integration of linkage maps for the Amphidiploid *Brassica napus* and comparative mapping with Arabidopsis and *Brassica rapa*.. BMC Genomics.

[55] Vezzulli S, Troggio M, Coppola G, Jermakow A, Cartwright D (2008). A reference integrated map for cultivated grapevine (*Vitis vinifera* L.) from three crosses, based on 283 SSR and 501 SNP-based markers.. Theor Appl Genet.

[56] Spiller M, Linde M, Hibrand-Saint Oyant L, Tsai C-J, Byrne D (2011). Towards a unified genetic map for diploid roses.. Theor Appl Genet.

[57] Salse J, Abrouk M, Bolot S, Guilhot N, Courcelle E (2009). Reconstruction of monocotelydoneous proto-chromosomes reveals faster evolution in plants than in animals.. Proc Natl Acad Sci, USA.

[58] Mace E, Jordan D (2011). Integrating sorghum whole genome sequence information with a compendium of sorghum QTL studies reveals uneven distribution of QTL and of gene-rich regions with significant implications for crop improvement.. Theor Appl Genet.

[59] Quraishi UM, Abrouk M, Murat F, Pont C, Foucrier S (2011). Cross-genome map based dissection of a nitrogen use efficiency ortho-metaQTL in bread wheat unravels concerted cereal genome evolution.. The Plant Journal.

[60] Muchero W, Ehlers J, Close T, Roberts P (2011). Genic SNP markers and legume synteny reveal candidate genes underlying QTL for *Macrophomina phaseolina* resistance and maturity in cowpea [Vigna unguiculata (L) Walp.].. BMC Genomics.

