# Supplementary material for: Saturation of an Intra-Gene Pool Linkage Map: Towards a Unified Consensus Linkage Map for Fine Mapping and Synteny Analysis in Common Bean
Source: PLoS One. 2011 Dec 8;6(12):e28135. doi: 10.1371/journal.pone.0028135 (PMC3234260; doi:10.1371/journal.pone.0028135)
Supplement: Table S1 — Summary of the markers evaluated in the DOR364×BAT477 (DB) population. (DOCX) [file pone.0028135.s001.docx]

Supplemental Table 1. Summary of the markers evaluated in the DOR364×BAT477 population.

| **Primer name** | **Reference** | **Type of marker** | **Genic/Genomic** | **Primers evaluated** | **Polymorphic primers** | **Polymorphism %** |
| --- | --- | --- | --- | --- | --- | --- |
| PVM | [1] | SSR-EST | Genic | 100 | 1 | 1.0 |
| BMd | Blair (unpublished) | SSR | Genic | 128 | 3 | 2.3 |
| BMe | Hurtado (unpublished) | SSR-EST | Genic | 106 | 7 | 6.6 |
| BMb | [2] | SSR-BES | Genomic | 116 | 10 | 8.6 |
| BMb | [3] | SSR-BES | Genomic | 114 | 7 | 6.1 |
| BMb | Cordoba (unpublished) | SSR-BES | Genomic | 325 | 26 | 8.0 |
| Genebank number | [4] | SSR | Genic | 20 | 0 | 0.0 |
| FJ | [5] | SSR | Genomic | 20 | 0 | 0.0 |
| PV | [6] | SSR | Genomic | 21 | 0 | 0.0 |
| Genebank number | [7] | SSR | Genomic | 21 | 0 | 0.0 |
| BMc | [8] | SSR | Genic | 120 | 3 | 2.5 |
| PV | [9] | SSR | Genic | 37 | 2 | 5.4 |
| BMc | [10]* | SSR | Genic | 258 | 12 | 4.7 |
| BMd | [11]* | SSR | Genic | 57 | 3 | 5.3 |
| PVM, FJ | [12] | SSR | Genic/Genomic | 80 | 4 | 5.0 |
| BM | [13]* | SSR | Genomic | 85 | 7 | 8.2 |
| IAC | [14] | SSR | Genomic | 123 | 12 | 9.8 |
| BMa, HRG, ME, PG2, GA, CAC | [15]* | SSR | Genomic | 85 | 14 | 16.5 |
| PVBR | [16] | SSR | Genomic | 98 | 22 | 22.4 |
| BM, GATS, AG | [17]* | SSR | Genomic | 68 | 19 | 27.9 |
| PVBR | [18] | SSR | Genomic | 20 | 8 | 40.0 |
| BSNP, g | [19,20] | EST-based markers (SSCP) | Genic | 393 | 6 | 1.5 |
| LEG | [21] | gene based marker (SSCP) | Genic | 111 | 2 | 1.8 |
| nod gene-BSn | Galeano (unpublished) | gene based marker (SSCP) | Genic | 200 | 3 | 1.5 |
| **Total** |  |  |  | **2,706** | **171** | **7.7** |
| * this markers were evaluated in [22] | | | | | | |

**Referencies**

1. Hanai L, Santini L, Camargo L, Fungaro M, Gepts P, et al. (2009) Extension of the core map of common bean with EST-SSR, RGA, AFLP, and putative functional markers. Molecular Breeding 25:25-45.

2. Córdoba JM, Chavarro C, Schlueter JA, Jackson SA, Blair MW (2010) Integration of physical and genetic maps of common bean through BAC-derived microsatellite markers. BMC Genomics 11:436.

3. Córdoba JM, Chavarro C, Rojas F, Muñoz C, Blair MW (2010) Identification and mapping of simple sequence repeat markers from common bean (*Phaseolus vulgaris* L.) bacterial artificial chromosome end sequences for genome characterization and genetic-physical map integration. Plant Genome 3: 154-165.

4. Guerra-Sanz JM (2004) Short Communication. New SSR markers of *Phaseolus vulgaris* from sequence databases. Plant Breeding 123: 87-89.

5. de Campos T, Benchimol LL, Moraes Carbonell SA, Chioratto AF, Fernandes Formighieri E, et al. (2007) Microsatellites for genetic studies and breeding programs in common bean. Pesq agropec bras 42: 589-592.

6. Caixeta ET, Borém A, Kelly JD (2005) Development of microsatellite markers based on BAC common bean clones. Crop Breeding and Applied Biotechnology 5: 125-133.

7. Yaish MWF, Perez de la Vega M (2003) Isolation of (GA)n microsatellite sequences and description of a predicted MADS-box sequence isolated from common bean (*Phaseolus vulgaris* L.). Genetics and Molecular Biology 26: 337-342.

8. Blair M, Hurtado N, Chavarro C, Munoz-Torres M, Giraldo M, et al. (2011) Gene-based SSR markers for common bean (*Phaseolus vulgaris* L.) derived from root and leaf tissue ESTs: an integration of the BMc series. BMC Plant Biology 11: 50.

9. Yu K, Park SJ, Poysa V, Gepts P (2000) Integration of simple sequence repeat (SSR) markers into a molecular linkage map of common bean (*Phaseolus vulgaris* L.). Journal of Heredity 91: 429-434.

10. Blair MW, Torres MM, Pedraza F, Giraldo MC, Buendia HF, et al. (2009) Development of microsatellite markers for common bean (*Phaseolus vulgaris* L.) based on screening of non-enriched, small-insert genomic libraries. Genome 52: 772-782.

11. Blair M, Pedraza F, Buendia H, Gaitan E, Beebe S, et al. (2003) Development of a genome wide anchored microsatellite for common bean (*Phaseolus vulgari*s L). Theor Appl Genet 107: 1362-1374.

12. Hanai LR, de Campos T, Aranha LE, Benchimol LL, Pereira de Souza A, et al. (2007) Development, characterization, and comparative analysis of polymorphism at common bean SSR loci isolated from genic and genomic sources. Genome 50: 266–277.

13. Blair MW, Torres MM, Giraldo MC, Pedraza F (2009) Development and diversity of Andean-derived, gene-based microsatellites for common bean (*Phaseolus vulgaris* L.). BMC Plant Biology 9.

14. Benchimol LL, de Campos T, Carbonell SAM, Colombo CA, Chioratto AF, et al. (2007) Structure of genetic diversity among common bean (*Phaseolus vulgaris* L.) varieties of Mesoamerican and Andean origins using new developed microsatellite markers. Genetic Resources and Crop Evolution 54: 1747-1762.

15. Blair MW, Buendía HF, Giraldo MC, Métais I, Peltier D (2008) Characterization of AT-rich microsatellites in common bean (*Phaseolus vulgaris L*.). Theor Appl Genet 118: 91-103.

16. Grisi MCM, Blair MW, Gepts P, Brondani C, Pereira PAA, et al. (2007) Genetic mapping of a new set of microsatellite markers in a reference common bean (*Phaseolus vulgaris*) population BAT93 x Jalo EEP558. Genetics and Molecular Research 6: 691-706.

17. Gáitan-Solis E, Duque MC, Edwards KJ, Tohme J (2002) Microsatellite Repeats in Common Bean (*Phaseolus vulgaris*): Isolation, Characterization, and Cross-Species Amplification in *Phaseolus* ssp. Crop Science 42: 2128–2136.

18. Buso GC, Amaral ZS, Brondani RV, Ferreira ME (2006) Microsatellite markers for the common bean *Phaseolus vulgaris*. Molecular Ecology Notes 6: 252–254.

19. Galeano CH, Fernandez AC, Gomez M, Blair MW (2009) Single strand conformation polymorphism based SNP and indel markers for genetic mapping and synteny analysis of common bean (*Phaseolus vulgaris* L.). BMC Genomics 10: 629.

20. McConnell M, Mamidi S, Lee R, Chikara S, Rossi M, et al. (2010) Syntenic relationships among legumes revealed using a gene-based genetic linkage map of common bean (*Phaseolus vulgaris* L.). Theor Appl Genet 121:1103-1116.

21. Hougaard BK, Madsen LH, Sandal N, Moretzsohn MC, Fredslund J, et al. (2008) Legume anchor markers link syntenic regions between *Phaseolus vulgaris*, *Lotus japonicus*, *Medicago truncatula* and *Arachis*. Genetics 119: 2299-2312.

22. Blair M, Galeano C, Tovar E, Muñoz Torres M, Castrillón A, et al. (2010) Development of a Mesoamerican intra-genepool genetic map for quantitative trait loci detection in a drought tolerant × susceptible common bean (*Phaseolus vulgaris* L.) cross. Molecular Breeding: 1-18.
